# Comment on: creating assessments as an active learning strategy: what are students’ perceptions? A mixed methods study

**DOI:** 10.1080/10872981.2019.1709277

**Published:** 2020-01-14

**Authors:** Alex Belshaw, Adam Mackie, Hannah Kay Phillips, Hannah Rodrigues, Andrew Russell

**Affiliations:** Medical School, University of Birmingham, Birmingham, UK

Dear Editor,

We read, with great interest, the article by Kurtz et al. [1] highlighting the potential benefits of teaching students to create assessments, involving multiple-choice questions (MCQs). As final year medical students attending the University of Birmingham, we have realised the all-important use of online tools, such as Peerwise, that enable students to construct, answer and share questions. We feel that the advantages of constructing and answering MCQs are under-recognised amongst medical students. Therefore, we welcome the recognition of this skill, outlined by Kurtz et al. [1] and suggest further discussion points.

Throughout our 5 years at university, we have used Peerwise to aid our revision by writing MCQs, which annually forms part of our assessment. Using this online tool we are able to formulate questions and receive instant feedback, an aspect of learning that Gibbs and Simpson [2] highlight as being of vital importance for students. Within the UK, many medical students adopt programmes such as Passmed and BMJ On Examination which are designed to replicate exam-style MCQs. This paper rightly suggests that students gain a greater understanding by posing questions and differentiating answers, a more thought-provoking process than MCQ practice.

Furthermore, only six MCQs were generated per student in this study whereas we create limitless MCQs, using this as an essential active revision tool. Although we use Peerwise to design MCQs, we have never received formal training. We believe that the benefit of training would adjust the way in which we approach MCQs, a finding that Kurtz et al. also notes in the ‘strategic test taking’ theme. Whilst some would argue that this is not the goal of examination, we agree that due to the complexity of MCQ-writing, not all questions are perfectly written. Therefore, identifying patterns in questioning results in students being less likely to select incorrect answers despite having the required knowledge.

Additionally, passive learning methods, such as memorising techniques, are often employed by students. However, we feel that these methods can be inefficient and fail to achieve a deeper understanding. Although this study found that MCQ-writing was a timely process, this form of active learning involves higher-order thinking leading to greater retention. Overall, we believe that active learning strategies have an important place in medical curricula and if our University was to incorporate training in MCQ-writing, alongside a greater emphasis on software such as Peerwise, this could prove invaluable to students learning.
